# Overview of occupational cancer in painters in Korea

**DOI:** 10.1186/s40557-018-0222-3

**Published:** 2018-02-06

**Authors:** Jun-Pyo Myong, Younmo Cho, Min Choi, Hyoung-Ryoul Kim

**Affiliations:** 1 Department of Occupational and Environmental Medicine, Seoul St. Mary’s Hospital, College of Medicine, the Catholic University of Korea, Banpo-daero 222, Seocho-gu, Seoul, 06591 Republic of Korea; 2 Center for Occupational and Environmental Medicine Seoul St. Mary’s Hospital, 222 Banpo-Daero Seocho-gu, Seoul, 06591 Republic of Korea

**Keywords:** Painter, Occupation, Cancer, Work-relatedness

## Abstract

Comprehensive consideration is necessary for setting guidelines to evaluate evidence of occupational cancer in painters due to work-related exposure to carcinogens in paint (a phenomenon termed herein as “work-relatedness”). The aim of the present research is to perform a comprehensive review and to suggest criteria for the provision of compensation for occupational neoplasm among painters in Korea. In order to perform a comprehensive review, this study assessed and evaluated scientific reports of carcinogenicities from the International Agency for Research on Cancer (IARC) and the Industrial Injuries Advisory Council (IIAC), as well as reviewed the existing literature about occupational exposure among painters in Korea and the epidemiologic investigations of claimed cases of cancer among painters in Korea. The IARC declares that occupational exposures in commercial painting are classified as Group 1 carcinogens for lung cancer and bladder cancer among painters. The epidemiologic studies show consistent causal relationships between occupational exposure in painters and cancers such as lung cancer [meta relative risk: 1.34 (95% confidence intervals (CIs): 1.23-1.41)] and bladder cancer [meta relative risk: 1.24 (95% CIs: 1.16-1.33)]. In reviewing occupational cancer risks for commercial painters, the Industrial Injuries Advisory Council (IIAC) confirms occupational cancer risks for lung and bladder cancer among commercial painters. According to the IIAC, however, the elevated cancer risks reported in existing literature are not doubled in either lung or bladder cancer in commercial painters relative to the risks of these cancers in the general population. Based on our review of existing Korean articles on the topic, painters are exposed to potential carcinogens including polycyclic aromatic hydrocarbons (PAHs), benzene, hexavalent chrome, crystalized silica, asbestos, and other agents, and relative levels are estimated within commercial painting processes. However, the cancer risks of occupational exposure to Group 1 carcinogens for lung and bladder cancer in painters per se are not fully assessed in existing Korean articles. Total work duration, potential carcinogens in paint, mixed exposure to paints across various industries such as construction and shipbuilding, exposure periods, latent periods, and other factors should be considered on an individual basis in investigating the work-relatedness of certain types of cancer in commercial painters.

## Background

In 1989, the International Agency for Research on Cancer (IARC) classified commercial painting as a cause of occupational exposure in painters to Group 1 carcinogens for lung and bladder cancer [1]. The IARC reaffirmed the increased risk of lung and bladder cancer among painters after verifying the conspiracy on potential carcinogens and work processes in commercial painting in 2010 [2]. In Korea, spray paint was included on a list of potential carcinogens in 2013. The Korea Occupational Safety and Health Agency (KOSHA) and Occupational Lung Diseases Institute have performed several epidemiologic investigations on lung cancer and hematologic malignancy among painters. The investigating teams have suggested that seven cases out of ten investigated cases demonstrate a positive relationship between painting processes and lung cancer.

Comprehensive consideration is necessary to establish guidelines for criteria to evaluate the work-relatedness of cancer risks in painters in Korea. These guidelines should be based on the most reasonable information presently available, taking into account epidemiologic research on the assessment of potential carcinogen exposure among painters in Korea and other countries, and compensation data in Korea. Until now, this type of comprehensive evaluation has not been performed in Korea. Therefore, the aim of the present research is to perform a comprehensive review and to suggest criteria for the provision of compensation for occupational neoplasm among painters in Korea.

## Review

### General characteristics of painting

Painting is the application of specific synthetic materials to the surfaces of products or buildings to protect the objects from corrosion and dirt or to generate cosmetic appeal [1, 2]. The general purposes of painting are protection and plastering. Electrical conduction, semi-conduction, contamination control, fire-retardation, temperature sensing, and magnetic painting are further classified as specific purposes of painting [1, 2].

Paint is comprised of various components with varying purposes. The components of paint are pigments and extenders (fillers), binders (resins), solvents, and additives. Pigments affect the color, viscosity, durability, and chemical properties of paint. Extenders are able to fill in gaps and improve the physical properties of coatings. The main roles of binders are to facilitate the hardening or adhesion of coatings. Solvents are used to mix the components of paint by dissolving binders. If painting is intended to meet specific purposes of construction, such as the application of biocides or ultraviolet stabilization, then additives are adapted. The typical components of paint are summarized in Table 1 [3].

**Table 1:** The category and type of major components in paints

Category		Major components
Pigments & fillers	Inorganic	Essential elements, clays, calcium carbonate, mica, silicas, talcs, titanium dioxide, and red iron oxide
	Organic	Azo pigments (Benzidine Yellow, etc.)
Binder & resins	Natural resins and oils	Rosin, vegetable and fish oils
	Synthetic resins	Cellulosic, phenolic, alkyl, vinyl, acrylic and methacrylic, polyesters and polyurethane resins, phthalic resins, chlorinated rubber derivatives, styrene-butadiene, silicone oils, and etc.
Additives	Surfactants & disperser	Lecithin, zinc or calcium naphthenate or octoate, oleates, oleic acid, polyphosphates, pyrophosphates, salts of arylalkyl-sulfonic acids and salts of polycarboxylic acids
	Driers	Metal salts of naphthenic acid (lead, calcium, cobalt, manganese, zirconium, zinc, cerium, lanthanum, and etc.), tall oil acid, 2-ethylhexanolic acid and neodecanoic acid, zirconium, calcium and cobalt-zirconium compounds
	Rheological additives	Gum arabic, gum tragacanth, starch, sodium alginate, methyl cellulose, hydroxyethyl cellulose, polyvinyl alcohol, ammonium caseinate, polyurethane derivates, polyacrylates, maleic anhydride copolymers, mineral fillers, magnesium montmorillonite clays, pyrogenic silicic acid, polyacrylamides, polyacrylic acid salts, and etc.
	Plasticizers	Dibutyl-, diethyl-, diethylhexyl- and dioctylphthalates, low molecularweight esters of adipic and sebacic acid, tributyl phosphate, castor oil, and polyester resins
	Biocides	Formaldehyde, isothiazolinones and chloroacetamide
	Antiskinning agent	Phenol derivatives, methoxyphenol,ortho-aminophenol, and polyhydroxyphenol
	Corrosion inhibitors	Red lead, zinc, chromium(III), aluminium, calcium and magnesium phosphates
	Nanoparticles	Titanium dioxide, silver or silver compounds, aluminium, oxide, fullerenes
	Light stabilizers	2-hydroxybenzophenones, 2-hydroxyphenylbenzitriazoles, oxalanilides, and 2-hydroxyphenyltriazines
Solvents		Petroleum and coal-tar distillates, alcohols, esters, ketones, glycols, synthesized glycol ethers and esters (mainly ethylene), and propylene glycol derivates

### Literature review of occupational exposure among painters in Korea

The Korean Journal of Occupational and Environmental Medicine (Annals of Occupational and Environmental Medicine), the Korean Journal of Preventive Medicine, the Korean Journal of Industrial Health, official reports from KOSHA, and existing epidemiologic investigation reports have been reviewed to estimate the previous exposure status of commercial painters to paint carcinogens in Korea. A total of 31 articles were reviewed and are summarized in Table 2.

**Table 2:** Literature review for potential exposure while painting process in Korea 1989-2010

Author Journal Published year	Industry (or occupation)	Exposure and/or biologic exposure indices (BEI)		Exposure dose GM (GSD or range)	Remark
Kim et al. [24] 1989	Chemical manufacture (23 places) Rubber manufacture(5) Basic metal manufacture(40) 1.painting 2.spray/dry Assembly metal product manufacture(112) Other manufacture(5)	Toluene, xylene Toluene, MEK Toluene N-hexane Toluene, MEK		25, 35 unit: ppm 75, 125 32(20-50) 55 24.5 (7.5-100), 66 (25-175)	Values without range included
Kim et al. [25] 1991	Machinery maintenance Painting (spray)	Toluene, urinary hippuric acid (HA)		20.73 (13.97) ppm, 0.52 (0.23) g/L	Toluene dose in air and Urinary HA
Kim et al. [26] 1991	Vehicle maintenance (4 paintings) Metal painting (7) Wood furniture painting (26) Instrument painting (7)			-organic solvent detection rate from thinner: Toluene (76.4%) - TLV excess By paintings: Instrument painting (71.4%) By components: PAHs (29.5%)	No comment for benzene Available information of paint property, type of organic solvents detected from thinner, and their exposure level.
Jeong et al. [27] 1991	Surveillance program from 1st Jan1990 ~ 31th Dec 1990 404 workers from 5 Painting shops included male more than 10 years : 62.7%, female 1-3 years 60.0%	Toluene MEK Acetone MIBK Methanol Xylene		48.3 (10-85) 151.6 (124-178) 13.7 (8-21) 100 (100) 20.5 (12-21) 60.0 (20-80)	
Lee et al. [28] 1992	Chemical fiber factory	Organic solvent, Urinary HA		2.953 (1.497), *p* < 0.01 unit: g/L	Relation between urinary MA and mental health
Kang et al. [29] 1993	-Low exposure: paint plant, instrument plant, leather plant -Medium exposure: running shoes plant, shoes plant -High exposure: shoes plant boat plant			Toluene level in air < 10 ppm 10-50 ppm > 50 ppm	
Kim et al. [30] 1993	Furniture painting, Metal painting	Toluene mixture	urinary NAG urinary MA	AM (SD), GM furniture painting(*n* = 33); metal painting(*n* = 18) Toluene: 30.1 (39.4), 18.3; 35.1 (21.0), 30.1 0.44 (0.55), 0.27; 0.46 (0.24). 0.41 Urinary NAG: 57.5 (31.11); 19.9 (19.7) Urinary MA: 567.1 (721.9); 462.4 (342.7) unit nmolMU/h of incubation/mg creatinine	no GM values of urinary NAG, urinary MA
Roh et al. [31] 1993	Vehicle manufacture (A), Auto mechanic (B)	m + p xylene, o- xylene	Urinary MA, urinary m + p xylene Urinary o-xylene	A (*n* = 151); B (*n* = 40) Unit (g/L) 0.36 (0.33); 0.29 (0.21) 0.09 (0.12); 0.03 (0.03) 0.21 (0.16); 0.06 (0.08)	Relation between organic solvent component and health effect
Lee et al. [32] 1995	Painting procedure of fishing rod manufacture	p-xylene m-xylene o-xylene toluene		3 places of painting a,b,c, 2.29 0.94 4.74 1.54 0.62 3.20 1.72 0.61 2.66 0.22 0.58 1.22	Surveillance by quantitative analysis of organic solvent
Choi et al. [4] 1997	Paint spray industry Metal manufacture Steel production for container manufacture usage	Total chrome Hexavalent chrome		Exposure time AM: 0.264, PM: 0.318 Automatic spray: 0.001-0.060, Manual spray: 0.029-0.226 mg/m3	Information of perforation of nasal septum
Hong et al. [33] 1997	Shipbuilding painting - spray (1-3) - brush (4-6)	Volatile coal tar pitch		Worker 1. Worker 2. Worker 3. 0.15 0.12 0.14 (unit mg/m^3^) Worker 4. Worker 5. Worker 6. 0.21 0.11 0.10 (unit mg/m^3^)	The relation between Coal tar included paint and phototoxic contact dermatitis
Paik et al. [34] 1998	Size of enterprise (large(L) medium (M), small (S)) (L) Elevator painting (17) (M) Vehicle painting(56) (S) Elevator painting (3) 244 samples 170 personal samples (PS) 74 areal samples (AS)	(L)Toluene, Xylene PS (*n* = 8) AS (*n* = 9) (M) n-hexane, toluene PS (*n* = 29) AS (*n* = 27) (S) Toluene, Xylene PS (*n* = 3)		Exposure index 0.006 (1.25) 0.005 (1.50) 0.01 (4.00) 0.002 (2.00) 0.35 (1.20)	
Shin et al. [5] 1999	Total 5 shipbuilding plant			Major organic solvent of paint, thinner, and binder ➔ xylene (60% of thinner, average: 67.1%) others: toluene, isopropanol, 2-metoxypropanol etc., xylene: included in every types of paints toluene: amino included paint 10-20%, epoxy included paint 13.6%, vinyl included paint 14.3% coal tar pitch: 40 types of paints included (13%) lead chromate, zinc potassium chromate included paint: 8% vehicle: epoxy resin(19.9%, mostly) > alkyd resin(16%) > acryl resin(14.2%)	Hazardous components of shipbuilding paint Presentation of silica exposure silica (silicon doxide) included in extender of 27 out of 309 paints (8.8%)
Won et al. [35] 1999	Metal manufacture 862 place, auto or ship mechanic 485 Electro device manufacture 454, chemical material manufacture 293 small company less than 50 workers	Organic solvent 54 types = > type 1 organic solvent 5 types, type 2 organic solvent 31, type 3 organic solvent 2+, non -legal measurement duty material 15, benzene Highest detection rate: toluene (84.8%) > xylene (46.4%) > methyl ethyl ketone (31.1%) > n-hexane 22.7%) > benzene (20.4%)		Organic solvent areal air sample Total work hour in the organic solvent handling workplace: average 505 (8.4) (480-720 min) Average time due to organic solvent usage 437 (28.7) (100-720 min) practical estimate time of organic solvent: average 254 (288.8) (40-382 min)	
Won et al. [36] 2000	Organic solvent 3280 work places 4181 work process			Highest frequency of all work processes spray: ketone: 0.85 times, ester 0.66 brush: Ketone 0.72, Aliphatic hydrocarbon 0.33 mixed: ketone 0.9, Alcohol 0.53	Detection number per single sample according to work process PAHs 1.14~ 2.39 times detected of all processes:
Joo et al. [37] 2000	Shipbuilding painting 674 workers	xyelne, ethyl benzene, ethyl toluene		17 ppm 4 ppm 3 ppm	
Koh et al. [38] 2001	Shipbuilding painting 28 workers By process -spray:10 workers -brush: 18 workers By sealing property -inside of the block -outside of the block -in/outside	xylene		12.81 (3.03) unit ppm 11.82 (2.94) - No. spray No. brush 6 41.68 (2.03) 27 15.49 (2.29) 3 5.16 (3.06) 9 2.77 (2.14) 21 10.38 (2.64) 18 16.78 (2.69)	Sample measurement 3 times relatively
Kwon et al. [39] 2001	Auto mechanic workplace (1)	- surfacer solvent based paint (*n* = 8) toluene, butyl acetate, m-xylene water based paint (*n* = 7) 2-butoxyl ethanol - top coating, color base,solvent based paint(*n* = 8) Butyl acetate, m-xylene, toluene - water based painting (*n* = 8) 2-butoxy ethanol		Unit ppm 27.76 (15.79-35.36), 21.82 (11.71-28.83), 10.9696 (5.96-14.34) 6.91 (5.73-7.92) 24.54 (11.56-32.59), 17.86 (8.50-23.73),14.88 (7.48-19.12) 4.72 (1.10-11.57)	*different components between solvent- and water-based paints: more diverse organic solvents included in solvent based paint
Kim et al. [40] 2001	Paint remove process	Methylene chloride		Personal sample(*n* = 14) 30.40 (3.39) Areal sample(*n* = 2) 2.24 unit ppm	Methylene chloride exposure of paint removal worker
Moon et al. [41] 2001	11 manufacture factories 1267 workers Painting process (442 workers, 34.2% of total workers)	1,2-Dichloroethane Cellosolve N.N.-dimethyl furan		2.0 (3.0) 3.0 (1.5) 5.5 (4.4) I-OHP level difference by process (u mol/mol creatinine)	Mixed solvents: used in 20 paint processes (average 12 types)
Jeon et al. [21] 2001	Coal tar pitch included paint Manufacture factory(*n* = 4) Shipbuilding plant(*n* = 4) Steel pipe plant (*n* = 2)	PAHs in air	1-OHP	Before/after work Shipbuilding: brush (*n* = 35) 12.66 (3.91)/29.06 (2.75) Steel pipe: paint (*n* = 14) 28.88 (6.80)/78.90 (3.18) Paint manufacture: mixing (*n* = 8) 1.83 (5.03)/1.96 (6.05) PAHs level by industry (mg/m^3^): Shipbuilding plant (*n* = 66) 0.092 (8.674) Steel pipe plant (*n* = 20) 0.520 (2.741) Paint manufacture (*n* = 25) 0.012 (3.685)	
Park et al. [42] 2002	Instrument factory(*n* = 3), furniture factory(*n* = 1), other material factory(*n* = 1): all painting process	MIBK, Toluene, Cellosolve acetate		0.251 (4.4318) 0.2442 (9.2979) 0.3872 (2.5435)	Air level organic solvent
Cho et al. [43] 2002	Auto mechanics (*n* = 23/ 54 workers) painting process polishing / spraying Average working year 11.4 yr. polishing 3.8 h/day spraying 2.1 h/day	dust lead	Serum Lead	(unit mg/m^3^) polishing 2.56 (0.73-10.13) 0.34 (0.44-0.91) spraying 0.93 (0.27-2.09) polishing 0.0021(N.D-0.0170) 0.0002 (N.D-0.0007) spraying 0.0009 (N.D-0.0056) 3.5 (1.3-19.7) 1.4 (N.D-5.7)	*serum lead: workers performed polishing and spraying by daily work condition ➔ no distribution by process
Kim et al. [22] 2005	Coal tar included paint process (*n* = 10) Exposure: 107 coal tar using workers Controls:201 office workers Coal tar paint used between 2001.05.29-2002.05.30	total PAHs	Urinary 1-OHP Naphtol	Exposure (*n* = 201) Before;after work (umol/mol creatinine) 8.89 (5.23); 19.02 (5.23) 26.34 (1.89); 33.08 (2.14) 120.17μg/m3(6862.36)	*smoking history+ Smoking and PAH co-exposure: effect of 1-OHP seems to change depending on the level of PAHs
Lee et al. [23] 2005	Auto mechanics putty process(*n* = 20, 43 workers: 2005.05-2005.09) working process of each workers depended on daily working condition (no regular process)	total 49 samples putty included toluene xylene n-buthyl acetate methyl isobutyl ketone methyl ethyl ketone(MEK) stylene	urinary MA, urinary MA, urinary mandelic acid	0.45 (0.50) 2.5 0.10 (0.30) 1.5 52.0 (46.3) 0.8 3.00 (2.45) 100 2.81 (2.26) 100 2.16 (1.59) 150 0.78 (0.42) 50 0.91 (0.29) 200 0.48 (0.50) 50	
Lee et al. [44] 2005	Paint manufacture (*n* = 5) (coal tar included) Steel pipe (*n* = 2) (painting of steel pipe after melting solid coal tar enamel) 44 workers	(PAHs) urinary I-OHP No. < 10 years 20 ≥ 10 years 24 paint manufacture 20 steel pipe 24		13.57 (9.413) 11.89 (6.823) 2.33 (4.409) 51.63 (3.144)	urinary I-OHP was 22 times high in workers of steel pipe painting than of paint manufacture. .
Kim et al. [45] 2006	301 lung cancer patients (2003.11-2004.11, admission in 4 Busan hospitals) *work related case	Exposure duration, exposure material Leather painter: 21 years, furniture painter : 10 years, chrome		1 case 1 case	Specific occupational lung cancer cases in Busan. *2 cases: both probable. *working environment mesasurement result was limited to access.
Min et al. [46] 2009	Shipbuilding painting process(spraying, brushing, paint equipment blasting, paint quality control)	MA Methyl MA MA Methyl MA MA Methyl MA		Spraying,brushing 0.228 (0.194) 0.263 (0.247) 0.279 (0.417) 0.228 (0.289) blasting 0.242 (0.250) 0.207 (0.182) 0.072 (0.144) 0.055 (0.114) Paint quality control 0.165 (0.137) 0.145 (0.467)	unit g/g creatinine
Sim et al. [47] 2009	Auto mechanic painting process	dust(*n* = 27) lead (*n* = 27) toluene(*n* = 27)		0.38 (1.78) 0.002 (2.29) 1.08 (2.76)	unit: ppm
Cho et al. [48] 2009	shipbuilding painter	Toleuene (100) xylene(100) methyl alcohol(200) MIBK (200)		Work environment measurement history In 1989, 1991, 1993, and 1994 70-80, 80-90, 18.15-19.399, and 0.41 100-110, 110, 34.737-56.411, and 68.85 70-80, −, −, and - -, 40-50, 1.535, and trace	Parkinson disease case report Workplace evaluation of the patient. unit: ppm
Lim et al. [20] 2010	128 lung cancer workers (1999-2005, epidemiologic survey of KOSHA) * work related case: 53 cases Painter included (3 cases, 5.7%)			Exposure duration /carcinogen/ lung cancer case 19.8 years (6.3-29.0)/ asbestos/ 33cases 18.7 (6.3-31.9)/ PAHs/ 23 21.4 9.0-40.0)/ chrome/ 17 20.5 (10.0-40.0)/ silica/ 14 (total number is more than 53 due to multiple causes of lung cancer)	*occupational lung cancer 53 case, non-occupational lung cancer 75 case: no significant difference among age, smoking history and cell type. (*P* > 0.05) *descripted exposure material and occupation respectively and no information available of connections of the two categories

The presence and relative levels of polycyclic aromatic hydrocarbons (PAHs), benzene, hexavalent chrome, crystalized silica, asbestos, and other carcinogenic agents have been examined and estimated in the context of commercial painting processes [3]. According to a 1995 report on the level of exposure to chrome in factories reporting patients with nasal septal perforation, the level of chrome exposure among the employed spray painters was below the permissible exposure limit (PEL) of 0.5 mg/m^3^ at recorded measurements of 0.246 mg/mg^3^ in the morning and 0.318 mg/m^3^ in the afternoon [4]. Research on exposure levels to hazardous materials in paints at five domestic shipyards in 1999 shows that lead chromate and zinc potassium chromate were detected in 8% of paints [5]. The component analysis of that research also reveals that silicon dioxides were detected in 27 samples (8.8%) of painting materials, including extender pigments. In other findings, the geometric means of exposure ranges of asbestos were 1.6 fibers/cm^3^ and 2.45 fibers/cm^3^ in automobile repair and ship repair processes, respectively [6]. However, asbestos remains undetected in the products of automobile manufacturing companies after 1998 [7].

### Scientific evidence for carcinogenicities

The IARC classifies the occupational exposures of commercial painting as Group 1 carcinogens for lung cancer and bladder cancer [1–3]. Existing epidemiologic studies show consistent causal relationships between occupational exposure in painters and cancers including lung and bladder cancer [3]. A meta-analysis that includes 17 cohort and linkage studies and 29 case-control studies shows that the meta-relative risk (meta-RR) for lung cancer is 1.34 (95% confidence intervals (CIs): 1.23-1.41) [3]. The results of additional meta-analysis including 11 cohort and record-linked studies and 28 case-control studies show a meta-RR for bladder cancer of 1.24 (95% CI: 1.16-1.33) [3]. However, the IARC does not assert that specific components of paints (such as chromate, PAH, benzene, and other agents) significantly increase the incidence or mortality from lung cancer or bladder cancer. The IARC indicates that no data on cancer in experimental animals are available [2]. The working group that has established a special section for “occupational exposure for painters” declares that occupational exposure hazards for painters per se include Group 1 carcinogens for lung and bladder cancer. In addition, the official report contains evidence of other relevant data about specific chemicals in common components of paint (e.g., cadmium, PAH, aromatic azo dyes, and other components) [2].

The Industrial Injuries Advisory Council (IIAC) for occupational cancer risks in commercial painters (among other industrial groups) is the official advisory council for assisting the UK government on prescribed industrial diseases [8]. The IIAC report includes a comprehensive review of epidemiologic data indicating occupational cancer risks and evaluating whether the risks for certain occupational cancers are more than doubled in painters compared to the general population [8]. The council also considers the study design of British doctors Doll and Hill in terms of their criteria on causation [9, 10] in epidemiologic studies published since 1972. The IIAC review team considers occupational cancer risks for lung and bladder cancer in commercial painters in particular (as opposed to the risks of these occupational cancers in paint manufacturers, for example) in the overall cohort study [8]. In fact, according to the literature, the elevated risks in occupational lung and bladder cancer in painters are not doubled in cases of either lung [11–19] or bladder cancer [14–19] relative to these risks in the general population. Reports of the IIAC specify that crucial confounding factors, such as smoking, might be one reason for the elevated incidence of lung and bladder cancer among painters.

### Epidemiologic investigation of claimed cases in Korea

Epidemiologic investigation for the work-relatedness of lung cancer in commercial painters in Korea has been performed in a total of 10 cases (Table 3). Seven painters were approved by investigation board in KOSHA. Significant exposure to potential carcinogens such as hexavalent chromate, asbestos, and crystalized silica has been provided as evidence of the work-relatedness of occupational cancers including lung and bladder cancer in commercial painters.

**Table 3:** The epidemiologic investigation for the work-relatedness by KOSHA and Occupational Lung Diseases Institute from 2000 to 2012

Deliberate organization	Diagnosis year	age/sex	Industry	Painting work duration (year)	Incubation period(year)	Exposed carcinogen	Approval	Specific remarks
KOSHA	2000	53/F	Shipbuilding	14	14	Coal tar (exposed to PAH)	yes	PAH exposure confirmed
KOSHA	2000	46/M	Vehicle manufacture	12	12	Not confirmed	no	5 years of printing history before painting
KOSHA	2001	39/M	Shipbuilding	7	14	PAH, silica	yes	7 years of grinding after painting in shipbuilding industry
KOSHA	2001	56/M	Home appliance painting	22	22	Not confirmed	no	Hexavalent chrome etc. not confirmed in the paint
KOSHA	2004	45/M	Vehicle manufacture	19	19	Not confirmed	no	
KOSHA	2006	45/M	Auto mechanics	26	26	Hexavalent chrome	yes	Hexavalent chrome confirmed in the paint
OLDI	2010	54/M	Shipbuilding, heavy industry	21	21	crystal quartz	yes	crystal quartz 1.3%-36.9% included in the paint
OLDI	2010	45/M	Vehicle manufacture	15	15	Hexavalent chrome	yes	Bumper polishing,painting: Hexavalent chrome 118.33μg/m3
OLDI	2011	63/M	Metal manufacture	10	10	Zinc chromate	yes	Possibly asbestos included in the filler, Possibility of silica, hexavalent chrome exposure
OLDI	2012	57/M	Boiler manufacture	26	26	Not confirmed	yes	Possibility of welding fume asbestos co-exposure

*KOSHA* Korean Occupational Safety and Health Agency

*OLDI* Occupational Lung Diseases Institute

## Discussion

### Issues for considering the work-relatedness of cancer in painters

Means of occupational exposure mainly involve the inhalation of gases and vapors from paint components (solvents, additives, pigment dust, and binders), as well as dermal absorption or ingestion [3]. The term professional painters typically does not include paint-product manufacturers or bystanders, but refers only to workers that brush or spray paint onto objects. In interpreting the job of commercial painting, several tasks are involved that should be defined in addition to the painting itself, including clean up and preparation. Accordingly, each task should be evaluated for potential exposures. Although painters engage in the entire process, the act of painting is regarded as the main means of exposure to various hazardous materials [3]. Based on the documentation of the IARC, occupational cancer is restricted to lung cancer and bladder cancer in the present review [1–3]. The IARC declares that the epidemiological evidence on occupational exposure in painters does not specify potential carcinogenic agents in paint [2]. Occupational exposure for painters encompasses the potential carcinogenic risks for lung cancer and bladder cancer. This perspective should be discussed in estimating the relationship between occupational exposure among painters and occupational cancer in Korea on an individual basis. Potential carcinogens, such as hexavalent chromate [4], asbestos [20], crystallized silica [5], and PAH from coal tar [21–23] are found in paint. In addition, exposures within specific industries (such as shipbuilding and construction) should be taken into account. Another consideration in evaluating exposure evidence is the period of exposure. Based on our literature review, coal tar, crystalized silica, and hexavalent chromate were used in workplace paints in Korea until late 1990 [4–6]. Up until the 2000s, the usage of coal tar paint was found in the metal industry [21–23]. Unfortunately, paint containing hexavalent chromate is still currently used in Korea.

## Conclusion

Established guidelines according to exposure periods, types of industry, and periodical features of the risks of occupational exposure for painters are currently undefined for occupational lung cancer and bladder cancer among painters in Korea. In addition, no country has defined specific guidelines for occupational cancer among painters. Therefore, total work duration, potential carcinogens in paint, mixed exposure to paints across industries such as construction and shipbuilding, exposure periods, latent periods, and other factors should be considered on an individual basis in investigating the work-relatedness of certain types of cancer in commercial painters.
